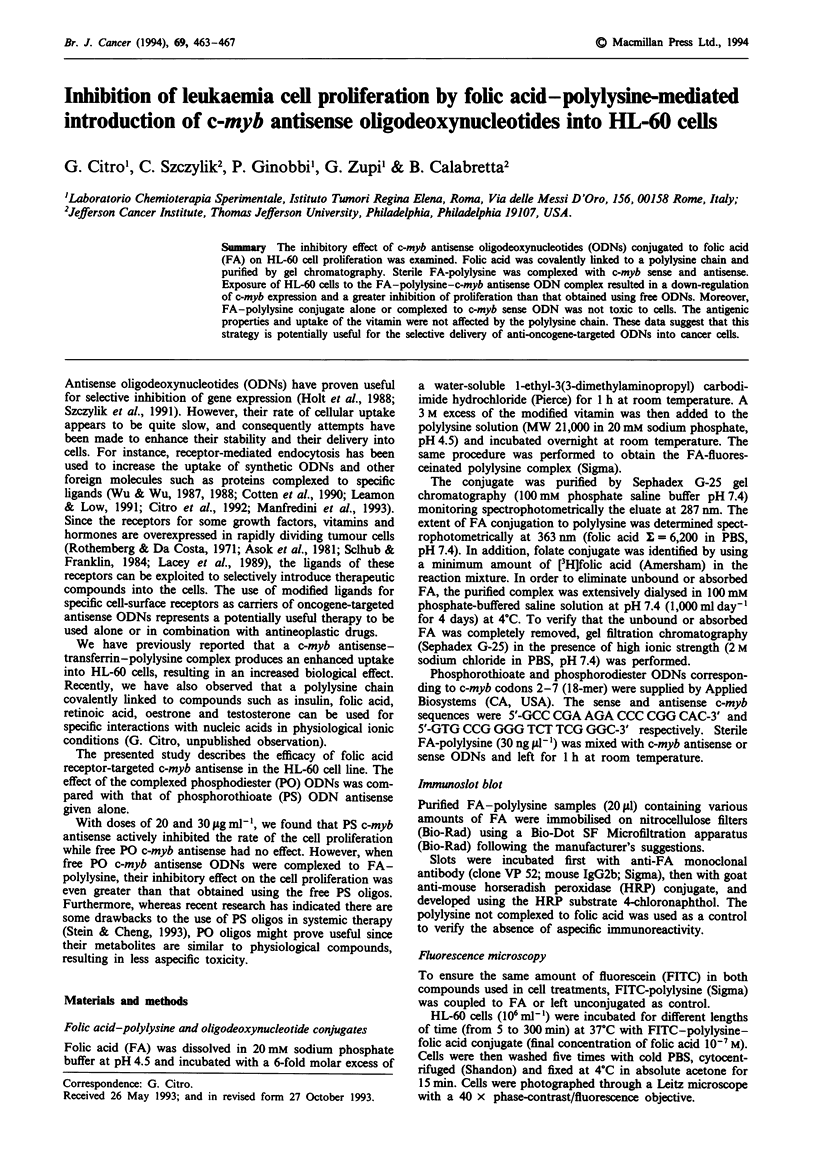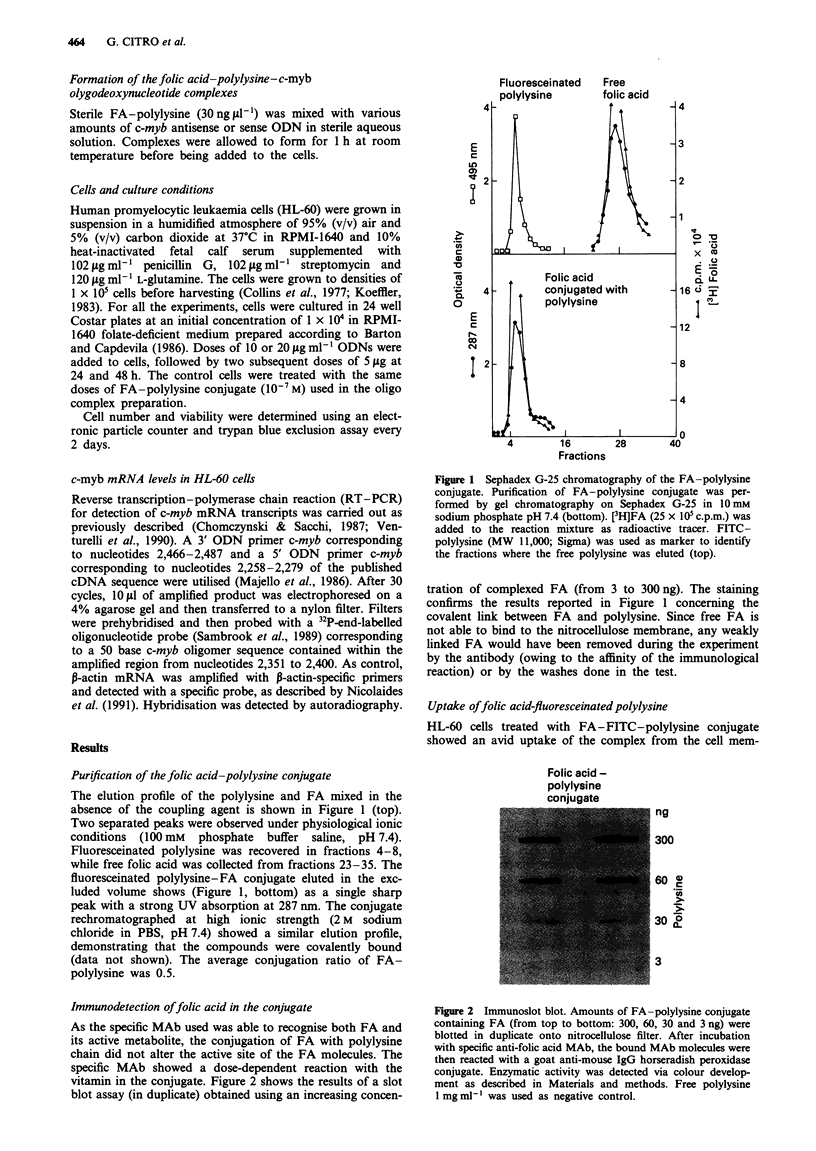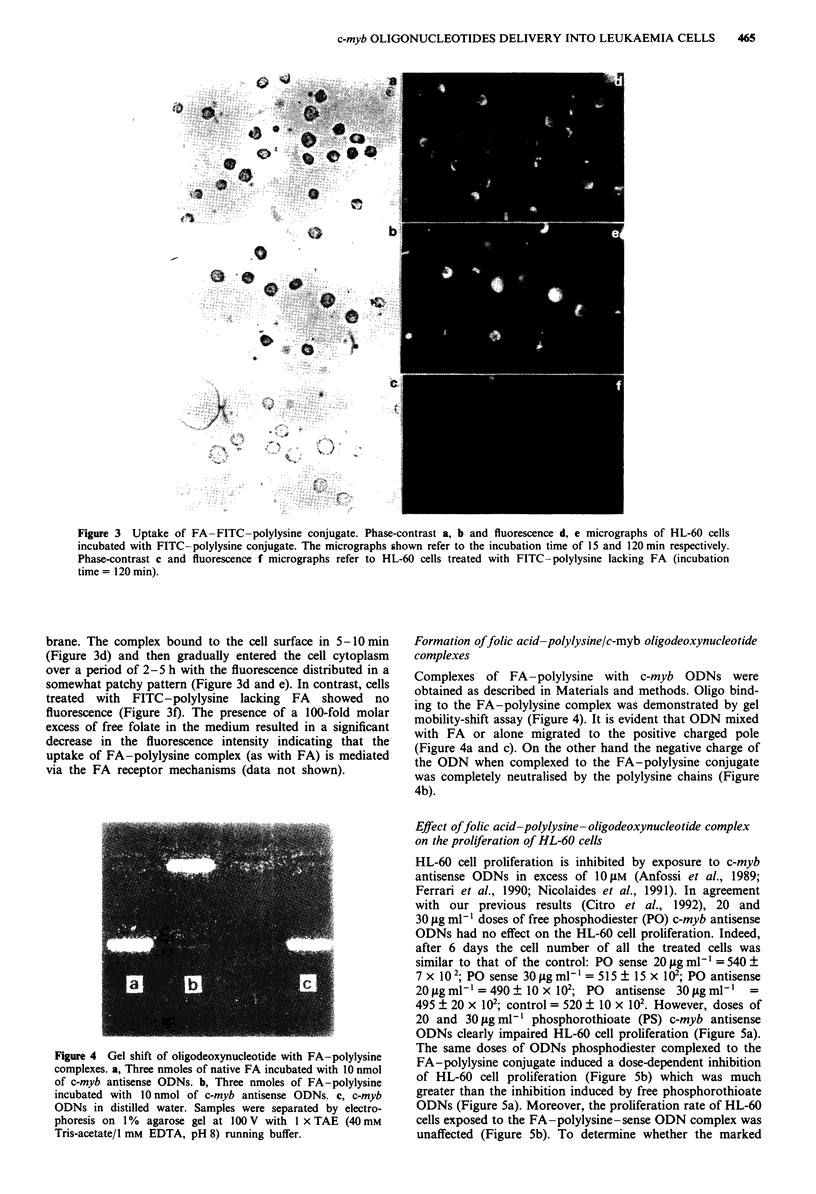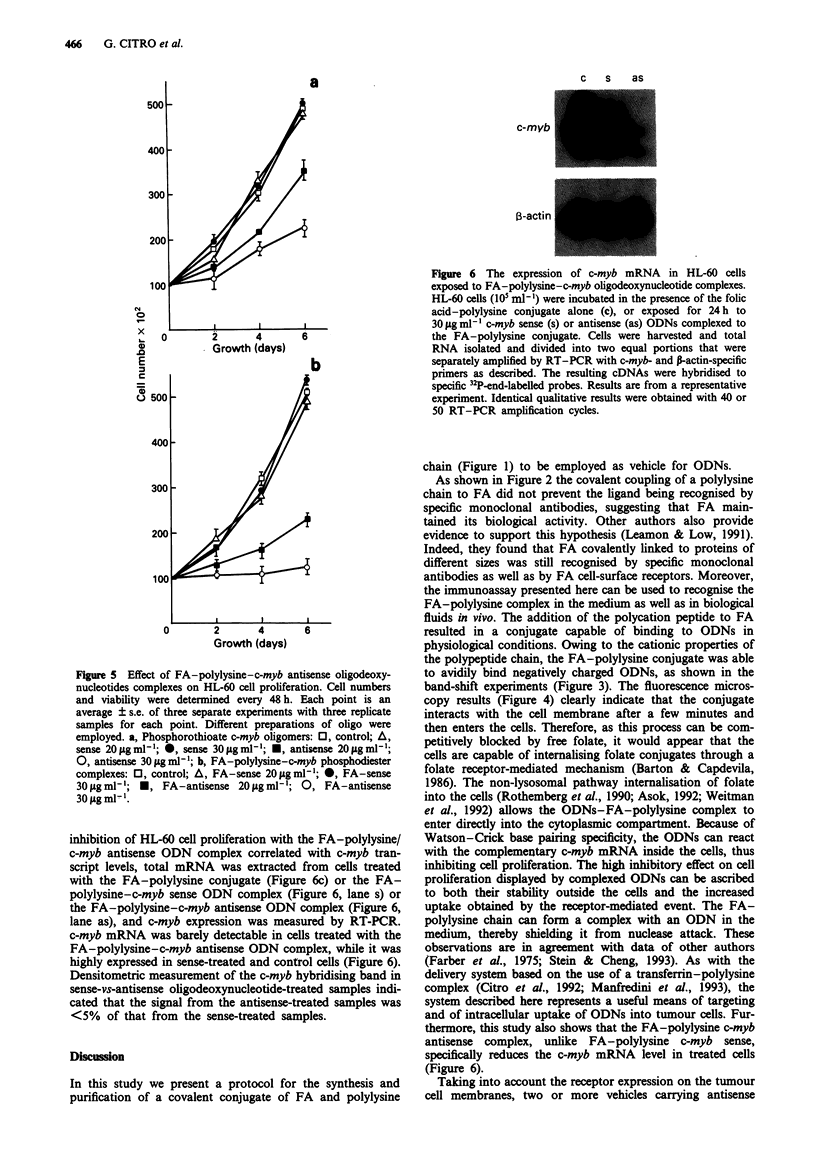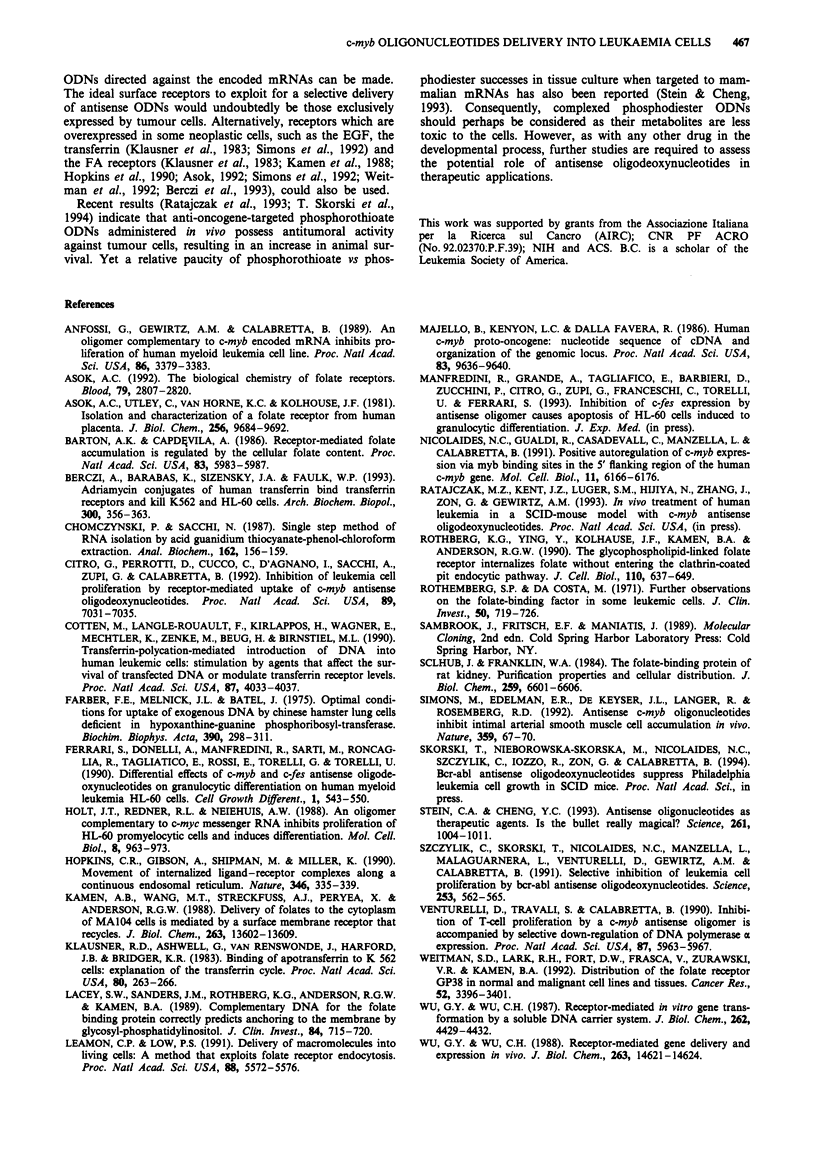# Inhibition of leukaemia cell proliferation by folic acid-polylysine-mediated introduction of c-myb antisense oligodeoxynucleotides into HL-60 cells.

**DOI:** 10.1038/bjc.1994.84

**Published:** 1994-03

**Authors:** G. Citro, C. Szczylik, P. Ginobbi, G. Zupi, B. Calabretta

**Affiliations:** Laboratorio Chemioterapia Sperimentale, Istituto Tumori Regina Elena, Roma, Italy.

## Abstract

**Images:**


Br. J. Cancer (1994), 69, 463–467 © Macmillan Press Ltd., 1994

### Inhibition of leukaemia cell proliferation by folic acid–polylysine-mediated introduction of c-*myb* antisense oligodeoxynucleotides into HL-60 cells

G. Citro[1], C. Szczylik[2], P. Ginobbi[1], G. Zupi[1] & B. Calabretta[2]

[1]*Laboratorio Chemioterapia Sperimentale, Istituto Tumori Regina Elena, Roma, Via delle Messi D'Oro, 156, 00158 Rome, Italy;* [2]*Jefferson Cancer Institute, Thomas Jefferson University, Philadelphia, Philadelphia 19107, USA.*

**Summary** The inhibitory effect of c-*myb* antisense oligodeoxynucleotides (ODNs) conjugated to folic acid (FA) on HL-60 cell proliferation was examined. Folic acid was covalently linked to a polylysine chain and purified by gel chromatography. Sterile FA-polylysine was complexed with c-*myb* sense and antisense. Exposure of HL-60 cells to the FA–polylysine–c-*myb* antisense ODN complex resulted in a down-regulation of c-*myb* expression and a greater inhibition of proliferation than that obtained using free ODNs. Moreover, FA–polylysine conjugate alone or complexed to c-*myb* sense ODN was not toxic to cells. The antigenic properties and uptake of the vitamin were not affected by the polylysine chain. These data suggest that this strategy is potentially useful for the selective delivery of anti-oncogene-targeted ODNs into cancer cells.

Antisense oligodeoxynucleotides (ODNs) have proven useful for selective inhibition of gene expression (Holt *et al.*, 1988; Szczylik *et al.*, 1991). However, their rate of cellular uptake appears to be quite slow, and consequently attempts have been made to enhance their stability and their delivery into cells. For instance, receptor-mediated endocytosis has been used to increase the uptake of synthetic ODNs and other foreign molecules such as proteins complexed to specific ligands (Wu & Wu, 1987, 1988; Cotten *et al.*, 1990; Leamon & Low, 1991; Citro *et al.*, 1992; Manfredini *et al.*, 1993). Since the receptors for some growth factors, vitamins and hormones are overexpressed in rapidly dividing tumour cells (Rothemberg & Da Costa, 1971; Asok *et al.*, 1981; Sclhub & Franklin, 1984; Lacey *et al.*, 1989), the ligands of these receptors can be exploited to selectively introduce therapeutic compounds into the cells. The use of modified ligands for specific cell-surface receptors as carriers of oncogene-targeted antisense ODNs represents a potentially useful therapy to be used alone or in combination with antineoplastic drugs.

We have previously reported that a c-*myb* antisense–transferrin–polylysine complex produces an enhanced uptake into HL-60 cells, resulting in an increased biological effect. Recently, we have also observed that a polylysine chain covalently linked to compounds such as insulin, folic acid, retinoic acid, oestrone and testosterone can be used for specific interactions with nucleic acids in physiological ionic conditions (G. Citro, unpublished observation).

The presented study describes the efficacy of folic acid receptor-targeted c-*myb* antisense in the HL-60 cell line. The effect of the complexed phosphodiester (PO) ODNs was compared with that of phosphorothioate (PS) ODN antisense given alone.

With doses of 20 and 30 $\mu g\ ml^{-1}$, we found that PS c-*myb* antisense actively inhibited the rate of the cell proliferation while free PO c-*myb* antisense had no effect. However, when free PO c-*myb* antisense ODNs were complexed to FA-polylysine, their inhibitory effect on the cell proliferation was even greater than that obtained using the free PS oligos. Furthermore, whereas recent research has indicated there are some drawbacks to the use of PS oligos in systemic therapy (Stein & Cheng, 1993), PO oligos might prove useful since their metabolites are similar to physiological compounds, resulting in less aspecific toxicity.

### Materials and methods

### *Folic acid–polylysine and oligodeoxynucleotide conjugates*

Folic acid (FA) was dissolved in 20 mM sodium phosphate buffer at pH 4.5 and incubated with a 6-fold molar excess of a water-soluble 1-ethyl-3(3-dimethylaminopropyl) carbodiimide hydrochloride (Pierce) for 1 h at room temperature. A 3 M excess of the modified vitamin was then added to the polylysine solution (MW 21,000 in 20 mM sodium phosphate, pH 4.5) and incubated overnight at room temperature. The same procedure was performed to obtain the FA-fluoresceinated polylysine complex (Sigma).

The conjugate was purified by Sephadex G-25 gel chromatography (100 mM phosphate saline buffer pH 7.4) monitoring spectrophotometrically the eluate at 287 nm. The extent of FA conjugation to polylysine was determined spectrophotometrically at 363 nm (folic acid $\Sigma = 6,200$ in PBS, pH 7.4). In addition, folate conjugate was identified by using a minimum amount of [$^3$H]folic acid (Amersham) in the reaction mixture. In order to eliminate unbound or absorbed FA, the purified complex was extensively dialysed in 100 mM phosphate-buffered saline solution at pH 7.4 (1,000 ml $day^{-1}$ for 4 days) at 4°C. To verify that the unbound or absorbed FA was completely removed, gel filtration chromatography (Sephadex G-25) in the presence of high ionic strength (2 M sodium chloride in PBS, pH 7.4) was performed.

Phosphorothioate and phosphorodiester ODNs corresponding to c-*myb* codons 2–7 (18-mer) were supplied by Applied Biosystems (CA, USA). The sense and antisense c-*myb* sequences were 5'-GCC CGA AGA CCC CGG CAC-3' and 5'-GTG CCG GGG TCT TCG GGC-3' respectively. Sterile FA-polylysine (30 ng $\mu l^{-1}$) was mixed with c-*myb* antisense or sense ODNs and left for 1 h at room temperature.

### *Immunoslot blot*

Purified FA–polylysine samples (20 µl) containing various amounts of FA were immobilised on nitrocellulose filters (Bio-Rad) using a Bio-Dot SF Microfiltration apparatus (Bio-Rad) following the manufacturer's suggestions.

Slots were incubated first with anti-FA monoclonal antibody (clone VP 52; mouse IgG2b; Sigma), then with goat anti-mouse horseradish peroxidase (HRP) conjugate, and developed using the HRP substrate 4-chloronaphthol. The polylysine not complexed to folic acid was used as a control to verify the absence of aspecific immunoreactivity.

### *Fluorescence microscopy*

To ensure the same amount of fluorescein (FITC) in both compounds used in cell treatments, FITC-polylysine (Sigma) was coupled to FA or left unconjugated as control.

HL-60 cells ($10^6\ ml^{-1}$) were incubated for different lengths of time (from 5 to 300 min) at 37°C with FITC–polylysine–folic acid conjugate (final concentration of folic acid $10^{-7}$ M). Cells were then washed five times with cold PBS, cytocentrifuged (Shandon) and fixed at 4°C in absolute acetone for 15 min. Cells were photographed through a Leitz microscope with a 40 × phase-contrast/fluorescence objective.

Correspondence: G. Citro.
Received 26 May 1993; and in revised form 27 October 1993.

*Formation of the folic acid–polylysine–c-*myb *olygodeoxynucleotide complexes*

Sterile FA–polylysine (30 ng $\mu l^{-1}$) was mixed with various amounts of c-*myb* antisense or sense ODN in sterile aqueous solution. Complexes were allowed to form for 1 h at room temperature before being added to the cells.

*Cells and culture conditions*

Human promyelocytic leukaemia cells (HL-60) were grown in suspension in a humidified atmosphere of 95% (v/v) air and 5% (v/v) carbon dioxide at 37°C in RPMI-1640 and 10% heat-inactivated fetal calf serum supplemented with 102 $\mu g\ ml^{-1}$ penicillin G, 102 $\mu g\ ml^{-1}$ streptomycin and 120 $\mu g\ ml^{-1}$ L-glutamine. The cells were grown to densities of $1 \times 10^5$ cells before harvesting (Collins *et al.*, 1977; Koeffler, 1983). For all the experiments, cells were cultured in 24 well Costar plates at an initial concentration of $1 \times 10^4$ in RPMI-1640 folate-deficient medium prepared according to Barton and Capdevila (1986). Doses of 10 or 20 $\mu g\ ml^{-1}$ ODNs were added to cells, followed by two subsequent doses of 5 $\mu g$ at 24 and 48 h. The control cells were treated with the same doses of FA–polylysine conjugate ($10^{-7}$ M) used in the oligo complex preparation.

Cell number and viability were determined using an electronic particle counter and trypan blue exclusion assay every 2 days.

*c-*myb *mRNA levels in HL-60 cells*

Reverse transcription–polymerase chain reaction (RT–PCR) for detection of c-*myb* mRNA transcripts was carried out as previously described (Chomczynski & Sacchi, 1987; Venturelli *et al.*, 1990). A 3′ ODN primer c-*myb* corresponding to nucleotides 2,466–2,487 and a 5′ ODN primer c-*myb* corresponding to nucleotides 2,258–2,279 of the published cDNA sequence were utilised (Majello *et al.*, 1986). After 30 cycles, 10 $\mu l$ of amplified product was electrophoresed on a 4% agarose gel and then transferred to a nylon filter. Filters were prehybridised and then probed with a $^{32}P$-end-labelled oligonucleotide probe (Sambrook *et al.*, 1989) corresponding to a 50 base c-*myb* oligomer sequence contained within the amplified region from nucleotides 2,351 to 2,400. As control, β-actin mRNA was amplified with β-actin-specific primers and detected with a specific probe, as described by Nicolaides *et al.* (1991). Hybridisation was detected by autoradiography.

### Results

*Purification of the folic acid–polylysine conjugate*

The elution profile of the polylysine and FA mixed in the absence of the coupling agent is shown in Figure 1 (top). Two separated peaks were observed under physiological ionic conditions (100 mM phosphate buffer saline, pH 7.4). Fluoresceinated polylysine was recovered in fractions 4–8, while free folic acid was collected from fractions 23–35. The fluoresceinated polylysine–FA conjugate eluted in the excluded volume shows (Figure 1, bottom) as a single sharp peak with a strong UV absorption at 287 nm. The conjugate rechromatographed at high ionic strength (2 M sodium chloride in PBS, pH 7.4) showed a similar elution profile, demonstrating that the compounds were covalently bound (data not shown). The average conjugation ratio of FA–polylysine was 0.5.

*Immunodetection of folic acid in the conjugate*

As the specific MAb used was able to recognise both FA and its active metabolite, the conjugation of FA with polylysine chain did not alter the active site of the FA molecules. The specific MAb showed a dose-dependent reaction with the vitamin in the conjugate. Figure 2 shows the results of a slot blot assay (in duplicate) obtained using an increasing concentration of complexed FA (from 3 to 300 ng). The staining confirms the results reported in Figure 1 concerning the covalent link between FA and polylysine. Since free FA is not able to bind to the nitrocellulose membrane, any weakly linked FA would have been removed during the experiment by the antibody (owing to the affinity of the immunological reaction) or by the washes done in the test.

**Figure 1** Sephadex G-25 chromatography of the FA–polylysine conjugate. Purification of FA–polylysine conjugate was performed by gel chromatography on Sephadex G-25 in 10 mM sodium phosphate pH 7.4 (bottom). [$^3$H]FA ($25 \times 10^5$ c.p.m.) was added to the reaction mixture as radioactive tracer. FITC–polylysine (MW 11,000; Sigma) was used as marker to identify the fractions where the free polylysine was eluted (top).

*Uptake of folic acid-fluoresceinated polylysine*

HL-60 cells treated with FA–FITC–polylysine conjugate showed an avid uptake of the complex from the cell mem-

**Figure 2** Immunoslot blot. Amounts of FA–polylysine conjugate containing FA (from top to bottom: 300, 60, 30 and 3 ng) were blotted in duplicate onto nitrocellulose filter. After incubation with specific anti-folic acid MAb, the bound MAb molecules were then reacted with a goat anti-mouse IgG horseradish peroxidase conjugate. Enzymatic activity was detected via colour development as described in Materials and methods. Free polylysine 1 mg $ml^{-1}$ was used as negative control.

**Figure 3** Uptake of FA–FITC–polylysine conjugate. Phase-contrast **a**, **b** and fluorescence **d**, **e** micrographs of HL-60 cells incubated with FITC–polylysine conjugate. The micrographs shown refer to the incubation time of 15 and 120 min respectively. Phase-contrast **c** and fluorescence **f** micrographs refer to HL-60 cells treated with FITC–polylysine lacking FA (incubation time = 120 min).

brane. The complex bound to the cell surface in 5–10 min (Figure 3d) and then gradually entered the cell cytoplasm over a period of 2–5 h with the fluorescence distributed in a somewhat patchy pattern (Figure 3d and e). In contrast, cells treated with FITC–polylysine lacking FA showed no fluorescence (Figure 3f). The presence of a 100-fold molar excess of free folate in the medium resulted in a significant decrease in the fluorescence intensity indicating that the uptake of FA–polylysine complex (as with FA) is mediated via the FA receptor mechanisms (data not shown).

**Figure 4** Gel shift of oligodeoxynucleotide with FA–polylysine complexes. **a**, Three nmoles of native FA incubated with 10 nmol of c-*myb* antisense ODNs. **b**, Three nmoles of FA–polylysine incubated with 10 nmol of c-*myb* antisense ODNs. **c**, c-*myb* ODNs in distilled water. Samples were separated by electrophoresis on 1% agarose gel at 100 V with 1 × TAE (40 mM Tris-acetate/1 mM EDTA, pH 8) running buffer.

### *Formation of folic acid–polylysine/c-*myb *oligodeoxynucleotide complexes*

Complexes of FA–polylysine with c-*myb* ODNs were obtained as described in Materials and methods. Oligo binding to the FA–polylysine complex was demonstrated by gel mobility-shift assay (Figure 4). It is evident that ODN mixed with FA or alone migrated to the positive charged pole (Figure 4a and c). On the other hand the negative charge of the ODN when complexed to the FA–polylysine conjugate was completely neutralised by the polylysine chains (Figure 4b).

### *Effect of folic acid–polylysine–oligodeoxynucleotide complex on the proliferation of HL-60 cells*

HL-60 cell proliferation is inhibited by exposure to c-*myb* antisense ODNs in excess of 10 μM (Anfossi *et al.*, 1989; Ferrari *et al.*, 1990; Nicolaides *et al.*, 1991). In agreement with our previous results (Citro *et al.*, 1992), 20 and 30 $\mu g\ ml^{-1}$ doses of free phosphodiester (PO) c-*myb* antisense ODNs had no effect on the HL-60 cell proliferation. Indeed, after 6 days the cell number of all the treated cells was similar to that of the control: PO sense 20 $\mu g\ ml^{-1} = 540 \pm 7 \times 10^2$; PO sense 30 $\mu g\ ml^{-1} = 515 \pm 15 \times 10^2$; PO antisense 20 $\mu g\ ml^{-1} = 490 \pm 10 \times 10^2$; PO antisense 30 $\mu g\ ml^{-1} = 495 \pm 20 \times 10^2$; control $= 520 \pm 10 \times 10^2$. However, doses of 20 and 30 $\mu g\ ml^{-1}$ phosphorothioate (PS) c-*myb* antisense ODNs clearly impaired HL-60 cell proliferation (Figure 5a). The same doses of ODNs phosphodiester complexed to the FA–polylysine conjugate induced a dose-dependent inhibition of HL-60 cell proliferation (Figure 5b) which was much greater than the inhibition induced by free phosphorothioate ODNs (Figure 5a). Moreover, the proliferation rate of HL-60 cells exposed to the FA–polylysine–sense ODN complex was unaffected (Figure 5b). To determine whether the marked

**Figure 5** Effect of FA-polylysine-c-*myb* antisense oligodeoxynucleotides complexes on HL-60 cell proliferation. Cell numbers and viability were determined every 48 h. Each point is an average ± s.e. of three separate experiments with three replicate samples for each point. Different preparations of oligo were employed. **a**, Phosphorothioate c-*myb* oligomers: □, control; △, sense 20 µg ml$^{-1}$; ●, sense 30 µg ml$^{-1}$; ■, antisense 20 µg ml$^{-1}$; ○, antisense 30 µg ml$^{-1}$; **b**, FA-polylysine-c-*myb* phosphodiester complexes: □, control; △, FA-sense 20 µg ml$^{-1}$; ●, FA-sense 30 µg ml$^{-1}$; ■, FA-antisense 20 µg ml$^{-1}$; ○, FA-antisense 30 µg ml$^{-1}$.

**Figure 6** The expression of c-*myb* mRNA in HL-60 cells exposed to FA-polylysine-c-*myb* oligodeoxynucleotide complexes. HL-60 cells ($10^5$ ml$^{-1}$) were incubated in the presence of the folic acid-polylysine conjugate alone (c), or exposed for 24 h to 30 µg ml$^{-1}$ c-*myb* sense (s) or antisense (as) ODNs complexed to the FA-polylysine conjugate. Cells were harvested and total RNA isolated and divided into two equal portions that were separately amplified by RT-PCR with c-*myb*- and β-actin-specific primers as described. The resulting cDNAs were hybridised to specific $^{32}$P-end-labelled probes. Results are from a representative experiment. Identical qualitative results were obtained with 40 or 50 RT-PCR amplification cycles.

inhibition of HL-60 cell proliferation with the FA-polylysine/c-*myb* antisense ODN complex correlated with c-*myb* transcript levels, total mRNA was extracted from cells treated with the FA-polylysine conjugate (Figure 6c) or the FA-polylysine-c-*myb* sense ODN complex (Figure 6, lane s) or the FA-polylysine-c-*myb* antisense ODN complex (Figure 6, lane as), and c-*myb* expression was measured by RT-PCR. c-*myb* mRNA was barely detectable in cells treated with the FA-polylysine-c-*myb* antisense ODN complex, while it was highly expressed in sense-treated and control cells (Figure 6). Densitometric measurement of the c-*myb* hybridising band in sense-*vs*-antisense oligodeoxynucleotide-treated samples indicated that the signal from the antisense-treated samples was <5% of that from the sense-treated samples.

### Discussion

In this study we present a protocol for the synthesis and purification of a covalent conjugate of FA and polylysine chain (Figure 1) to be employed as vehicle for ODNs.

As shown in Figure 2 the covalent coupling of a polylysine chain to FA did not prevent the ligand being recognised by specific monoclonal antibodies, suggesting that FA maintained its biological activity. Other authors also provide evidence to support this hypothesis (Leamon & Low, 1991). Indeed, they found that FA covalently linked to proteins of different sizes was still recognised by specific monoclonal antibodies as well as by FA cell-surface receptors. Moreover, the immunoassay presented here can be used to recognise the FA-polylysine complex in the medium as well as in biological fluids *in vivo*. The addition of the polycation peptide to FA resulted in a conjugate capable of binding to ODNs in physiological conditions. Owing to the cationic properties of the polypeptide chain, the FA-polylysine conjugate was able to avidily bind negatively charged ODNs, as shown in the band-shift experiments (Figure 3). The fluorescence microscopy results (Figure 4) clearly indicate that the conjugate interacts with the cell membrane after a few minutes and then enters the cells. Therefore, as this process can be competitively blocked by free folate, it would appear that the cells are capable of internalising folate conjugates through a folate receptor-mediated mechanism (Barton & Capdevila, 1986). The non-lysosomal pathway internalisation of folate into the cells (Rothemberg *et al*., 1990; Asok, 1992; Weitman *et al*., 1992) allows the ODNs-FA-polylysine complex to enter directly into the cytoplasmic compartment. Because of Watson-Crick base pairing specificity, the ODNs can react with the complementary c-*myb* mRNA inside the cells, thus inhibiting cell proliferation. The high inhibitory effect on cell proliferation displayed by complexed ODNs can be ascribed to both their stability outside the cells and the increased uptake obtained by the receptor-mediated event. The FA-polylysine chain can form a complex with an ODN in the medium, thereby shielding it from nuclease attack. These observations are in agreement with data of other authors (Farber *et al*., 1975; Stein & Cheng, 1993). As with the delivery system based on the use of a transferrin-polylysine complex (Citro *et al*., 1992; Manfredini *et al*., 1993), the system described here represents a useful means of targeting and of intracellular uptake of ODNs into tumour cells. Furthermore, this study also shows that the FA-polylysine c-*myb* antisense complex, unlike FA-polylysine c-*myb* sense, specifically reduces the c-*myb* mRNA level in treated cells (Figure 6).

Taking into account the receptor expression on the tumour cell membranes, two or more vehicles carrying antisense

ODNs directed against the encoded mRNAs can be made. The ideal surface receptors to exploit for a selective delivery of antisense ODNs would undoubtedly be those exclusively expressed by tumour cells. Alternatively, receptors which are overexpressed in some neoplastic cells, such as the EGF, the transferrin (Klausner *et al.*, 1983; Simons *et al.*, 1992) and the FA receptors (Klausner *et al.*, 1983; Kamen *et al.*, 1988; Hopkins *et al.*, 1990; Asok, 1992; Simons *et al.*, 1992; Weitman *et al.*, 1992; Berczi *et al.*, 1993), could also be used.

Recent results (Ratajczak *et al.*, 1993; T. Skorski *et al.*, 1994) indicate that anti-oncogene-targeted phosphorothioate ODNs administered *in vivo* possess antitumoral activity against tumour cells, resulting in an increase in animal survival. Yet a relative paucity of phosphorothioate *vs* phosphodiester successes in tissue culture when targeted to mammalian mRNAs has also been reported (Stein & Cheng, 1993). Consequently, complexed phosphodiester ODNs should perhaps be considered as their metabolites are less toxic to the cells. However, as with any other drug in the developmental process, further studies are required to assess the potential role of antisense oligodeoxynucleotides in therapeutic applications.

This work was supported by grants from the Associazione Italiana per la Ricerca sul Cancro (AIRC); CNR PF ACRO (No. 92.02370:P.F.39); NIH and ACS. B.C. is a scholar of the Leukemia Society of America.

### References

ANFOSSI, G., GEWIRTZ, A.M. & CALABRETTA, B. (1989). An oligomer complementary to c-*myb* encoded mRNA inhibits proliferation of human myeloid leukemia cell line. *Proc. Natl Acad. Sci. USA*, **86**, 3379–3383.

ASOK, A.C. (1992). The biological chemistry of folate receptors. *Blood*, **79**, 2807–2820.

ASOK, A.C., UTLEY, C., VAN HORNE, K.C. & KOLHOUSE, J.F. (1981). Isolation and characterization of a folate receptor from human placenta. *J. Biol. Chem.*, **256**, 9684–9692.

BARTON, A.K. & CAPDEVILA, A. (1986). Receptor-mediated folate accumulation is regulated by the cellular folate content. *Proc. Natl Acad. Sci. USA*, **83**, 5983–5987.

BERCZI, A., BARABAS, K., SIZENSKY, J.A. & FAULK, W.P. (1993). Adriamycin conjugates of human transferrin bind transferrin receptors and kill K562 and HL-60 cells. *Arch. Biochem. Biopol.*, **300**, 356–363.

CHOMCZYNSKI, P. & SACCHI, N. (1987). Single step method of RNA isolation by acid guanidium thiocyanate-phenol-chloroform extraction. *Anal. Biochem.*, **162**, 156–159.

CITRO, G., PERROTTI, D., CUCCO, C., D'AGNANO, I., SACCHI, A., ZUPI, G. & CALABRETTA, B. (1992). Inhibition of leukemia cell proliferation by receptor-mediated uptake of c-*myb* antisense oligodeoxynucleotides. *Proc. Natl Acad. Sci. USA*, **89**, 7031–7035.

COTTEN, M., LANGLE-ROUAULT, F., KIRLAPPOS, H., WAGNER, E., MECHTLER, K., ZENKE, M., BEUG, H. & BIRNSTIEL, M.L. (1990). Transferrin-polycation-mediated introduction of DNA into human leukemic cells: stimulation by agents that affect the survival of transfected DNA or modulate transferrin receptor levels. *Proc. Natl Acad. Sci. USA*, **87**, 4033–4037.

FARBER, F.E., MELNICK, J.L. & BATEL, J. (1975). Optimal conditions for uptake of exogenous DNA by chinese hamster lung cells deficient in hypoxanthine-guanine phosphoribosyl-transferase. *Biochim. Biophys. Acta*, **390**, 298–311.

FERRARI, S., DONELLI, A., MANFREDINI, R., SARTI, M., RONCAGLIA, R., TAGLIATICO, E., ROSSI, E., TORELLI, G. & TORELLI, U. (1990). Differential effects of c-*myb* and c-*fes* antisense oligodeoxynucleotides on granulocytic differentiation on human myeloid leukemia HL-60 cells. *Cell Growth Different.*, **1**, 543–550.

HOLT, J.T., REDNER, R.L. & NEIEHUIS, A.W. (1988). An oligomer complementary to c-*myc* messenger RNA inhibits proliferation of HL-60 promyelocytic cells and induces differentiation. *Mol. Cell. Biol.*, **8**, 963–973.

HOPKINS, C.R., GIBSON, A., SHIPMAN, M. & MILLER, K. (1990). Movement of internalized ligand–receptor complexes along a continuous endosomal reticulum. *Nature*, **346**, 335–339.

KAMEN, A.B., WANG, M.T., STRECKFUSS, A.J., PERYEA, X. & ANDERSON, R.G.W. (1988). Delivery of folates to the cytoplasm of MA104 cells is mediated by a surface membrane receptor that recycles. *J. Biol. Chem.*, **263**, 13602–13609.

KLAUSNER, R.D., ASHWELL, G., VAN RENSWONDE, J., HARFORD, J.B. & BRIDGER, K.R. (1983). Binding of apotransferrin to K 562 cells: explanation of the transferrin cycle. *Proc. Natl Acad. Sci. USA*, **80**, 263–266.

LACEY, S.W., SANDERS, J.M., ROTHBERG, K.G., ANDERSON, R.G.W. & KAMEN, B.A. (1989). Complementary DNA for the folate binding protein correctly predicts anchoring to the membrane by glycosyl-phosphatidylinositol. *J. Clin. Invest.*, **84**, 715–720.

LEAMON, C.P. & LOW, P.S. (1991). Delivery of macromolecules into living cells: A method that exploits folate receptor endocytosis. *Proc. Natl Acad. Sci. USA*, **88**, 5572–5576.

MAJELLO, B., KENYON, L.C. & DALLA FAVERA, R. (1986). Human c-*myb* proto-oncogene: nucleotide sequence of cDNA and organization of the genomic locus. *Proc. Natl Acad. Sci. USA*, **83**, 9636–9640.

MANFREDINI, R., GRANDE, A., TAGLIAFICO, E., BARBIERI, D., ZUCCHINI, P., CITRO, G., ZUPI, G., FRANCESCHI, C., TORELLI, U. & FERRARI, S. (1993). Inhibition of c-*fes* expression by antisense oligomer causes apoptosis of HL-60 cells induced to granulocytic differentiation. *J. Exp. Med.* (in press).

NICOLAIDES, N.C., GUALDI, R., CASADEVALL, C., MANZELLA, L. & CALABRETTA, B. (1991). Positive autoregulation of c-*myb* expression via myb binding sites in the 5′ flanking region of the human c-*myb* gene. *Mol. Cell. Biol.*, **11**, 6166–6176.

RATAJCZAK, M.Z., KENT, J.Z., LUGER, S.M., HIJIYA, N., ZHANG, J., ZON, G. & GEWIRTZ, A.M. (1993). *In vivo* treatment of human leukemia in a SCID-mouse model with c-*myb* antisense oligodeoxynucleotides. *Proc. Natl Acad. Sci. USA*, (in press).

ROTHBERG, K.G., YING, Y., KOLHAUSE, J.F., KAMEN, B.A. & ANDERSON, R.G.W. (1990). The glycophospholipid-linked folate receptor internalizes folate without entering the clathrin-coated pit endocytic pathway. *J. Cell. Biol.*, **110**, 637–649.

ROTHEMBERG, S.P. & DA COSTA, M. (1971). Further observations on the folate-binding factor in some leukemic cells. *J. Clin. Invest.*, **50**, 719–726.

SAMBROOK, J., FRITSCH, E.F. & MANIATIS, J. (1989). *Molecular Cloning*, 2nd edn. Cold Spring Harbor Laboratory Press: Cold Spring Harbor, NY.

SCLHUB, J. & FRANKLIN, W.A. (1984). The folate-binding protein of rat kidney. Purification properties and cellular distribution. *J. Biol. Chem.*, **259**, 6601–6606.

SIMONS, M., EDELMAN, E.R., DE KEYSER, J.L., LANGER, R. & ROSEMBERG, R.D. (1992). Antisense c-*myb* oligonucleotides inhibit intimal arterial smooth muscle cell accumulation *in vivo*. *Nature*, **359**, 67–70.

SKORSKI, T., NIEBOROWSKA-SKORSKA, M., NICOLAIDES, N.C., SZCZYLIK, C., IOZZO, R., ZON, G. & CALABRETTA, B. (1994). Bcr-abl antisense oligodeoxynucleotides suppress Philadelphia leukemia cell growth in SCID mice. *Proc. Natl Acad. Sci.*, in press.

STEIN, C.A. & CHENG, Y.C. (1993). Antisense oligonucleotides as therapeutic agents. Is the bullet really magical? *Science*, **261**, 1004–1011.

SZCZYLIK, C., SKORSKI, T., NICOLAIDES, N.C., MANZELLA, L., MALAGUARNERA, L., VENTURELLI, D., GEWIRTZ, A.M. & CALABRETTA, B. (1991). Selective inhibition of leukemia cell proliferation by bcr-abl antisense oligodeoxynucleotides. *Science*, **253**, 562–565.

VENTURELLI, D., TRAVALI, S. & CALABRETTA, B. (1990). Inhibition of T-cell proliferation by a c-*myb* antisense oligomer is accompanied by selective down-regulation of DNA polymerase α expression. *Proc. Natl Acad. Sci. USA*, **87**, 5963–5967.

WEITMAN, S.D., LARK, R.H., FORT, D.W., FRASCA, V., ZURAWSKI, V.R. & KAMEN, B.A. (1992). Distribution of the folate receptor GP38 in normal and malignant cell lines and tissues. *Cancer Res.*, **52**, 3396–3401.

WU, G.Y. & WU, C.H. (1987). Receptor-mediated *in vitro* gene transformation by a soluble DNA carrier system. *J. Biol. Chem.*, **262**, 4429–4432.

WU, G.Y. & WU, C.H. (1988). Receptor-mediated gene delivery and expression *in vivo*. *J. Biol. Chem.*, **263**, 14621–14624.